# Prognosis of polymerase epsilon (*POLE*) mutation in high-grade endometrioid endometrial cancer: Systematic review and meta-analysis

**DOI:** 10.1016/j.ygyno.2024.01.018

**Published:** 2024-01-22

**Authors:** Joao Casanova, Gonçalo Silva Duarte, Ana Gomes da Costa, Ana Catarino, Mónica Nave, Telma Antunes, Sofia Silvério Serra, Sara Simões Dias, Nadeem Abu-Rustum, Jorge Lima

**Affiliations:** a Gynecologic Oncology Unit, Hospital da Luz Lisboa, Lisboa, Portugal; b Department of Obstetrics and Gynecology, LUZ SAÚDE, Hospital da Luz Lisboa, Lisboa, Portugal; c Laboratory of Clinical Pharmacology and Therapeutics, Faculty of Medicine, University of Lisbon, Lisboa, Portugal; d Hospital da Luz Lisboa, Lisboa, Portugal; e Department of Pathology, Hospital da Luz Lisboa, Lisboa, Portugal; f Department of Oncology, Hospital da Luz Lisboa, Lisboa, Portugal; g Department of Radiation Oncology, Hospital da Luz Lisboa, Lisboa, Portugal; h Library of NOVA Medical School, NMS, Universidade NOVA de Lisboa, Lisboa, Portugal; i CHRC, NOVA Medical School, Faculdade de Ciências Médicas, NMS, FCM, Universidade NOVA de Lisboa, Lisboa, Portugal; j ciTechCare-Center for Innovative Care and Health Technology, Polytechnic of Leiria, Leiria, Portugal; k Gynecologic Service, Department of Surgery, Memorial Sloan-Kettering Cancer Center, Weill Medical College of Cornell University, New York, USA

**Keywords:** Endometrial cancer, Endometrioid, High-grade, Meta-analysis, *POLE*, Prognosis, Systematic review

## Abstract

**Background.:**

*POLE* mutated endometrial carcinomas may represent a subspecific type of tumors harboring a more favorable prognosis. Grade 3 (G3 or high-grade) endometrioid endometrial carcinomas remain a clinical dilemma, with some tumors behaving as the low-grade counterparts and others presenting a more aggressive behavior.

**Objectives.:**

To determine the association between *POLE* mutational status and the overall-survival (OS) and progression-free-survival (PFS) of patients with G3 endometrioid endometrial cancer (EC). We also aimed to determine the prevalence of *POLE* mutations in G3 endometrioid EC.

**Methods.:**

We conducted a systematic review in accordance with the Preferred Reporting Items for Systematic Reviews and Meta-Analyses (PRISMA) guidelines (PROSPERO No: CRD4202340008). We searched the following electronic databases: PubMed/Medline, EMBASE, Cochrane Library, Scopus, and Web of Science. For time-to-event data, the effect of *POLE* mutation in G3 EC was described using hazard ratios (HRs) and corresponding 95% confidence intervals (CIs). Individual patient data for each study was investigated if available from the study authors. If individual patient data were not available, information regarding time-to-event outcomes was extracted using an appropriate methodology. OS and PFS were analyzed using both one-stage and two-stage approaches, the Kaplan-Meier method, and Cox-proportional hazards models.

**Results.:**

This systematic review and meta-analysis included 19 studies with 3092 patients who had high-grade endometrioid EC. Patients with *POLE* mutation*s* had lower risks of death (HR = 0.36, 95% CI 0.26 to 0.50, I^2^ = 0%, 10 trials) and disease progression (HR = 0.31, 95% CI 0.17 to 0.57, I^2^ = 33%, 10 trials). The pooled prevalence of *POLE* mutation was 11% (95% CI 9 to 13, I^2^ = 68%, 18 studies).

**Conclusion.:**

*POLE* mutations in high-grade endometrioid EC are associated with a more favorable prognosis with increased OS and PFS.

## 1. Introduction

Endometrial cancer (EC) is the most common gynecologic malignancy in Western countries, and its incidence and mortality rates are rising [1]. As obesity rates increase, the incidence of EC is also increasing in line with the obesity epidemic [1,2]. Similar to other malignancies, the risk of EC is strongly associated with obesity, increasing 50% for each 5-unit increase in the body mass index (BMI) [3].

In the spectrum of metabolic syndrome, diabetes is commonly associated with the risk of EC [4]. Conditions associated with high estrogen levels are also well-known risk factors for EC [1]. Furthermore, early menstruation, late menopause, estrogen therapy, estrogen-producing tumors, and polycystic ovarian syndrome (PCOS) are all conditions associated with increased risk of EC [5]. Tamoxifen, a drug with antiestrogenic effects in breast tissue and proestrogenic effects in the uterus, is also associated with a two-fold increase in the risk of EC [6].

EC is a malignancy that predominantly affects post-menopausal women (the average age of diagnosis is 63 years), but data has been showing an increase among women under 50 years of age [1]. Thus, younger women with a recent diagnosis of EC should be considered for Lynch syndrome testing as this genetic syndrome involves a lifetime risk of EC of 40–60% [7]. Classically, EC has been classified as type I (hormone-dependent) and type II (hormone-independent) tumors. Type I tumors have endometrioid histology and comprise roughly 80% of all EC cases. Type II tumors have non-endometrioid histology and include serous, clear-cell, and carcinosarcoma morphologies [1].

Tumors are graded according to the International Federation of Obstetrics and Gynecology (FIGO) system based on endometrioid histology. This system uses a scale of 1 to 3 and refers to the ratio of glandular to solid-tumor elements [8]. Focusing on endometrioid histology, grade 1 (G1) and grade 2 (G2) tumors (low-grade tumors) usually have a favorable prognosis, whereas grade 3 (G3) tumors (high-grade) are associated with a heterogenous prognosis, sometimes resembling that of non-endometrioid EC. Thus, G3 endometrioid EC tumors can be considered somewhat of a clinical and pathological conundrum: clinically, they can behave similarly to the most aggressive non-endometrioid EC subtypes or present a good prognosis resembling that of low-grade EC [9]. The histological diagnosis is also controversial as it is associated with interobserver variability and poor reproducibility [9].

In a landmark paper published in 2013, the Cancer Genome Atlas (TCGA) opened new fields of research, changed the landscape of EC, and paved the way for a more tailored approach to this malignancy [10]. Based on genome-wide analysis, the TCGA consortium concluded that EC could be divided in four molecular subtypes. The first is the ultramutated group, which is defined by mutations in the exonuclease domain of the polymerase epsilon (*POLE*) gene. The second is the micro-satellite unstable subgroup, which involves deficiency in one or more mismatch repair proteins (MMRd), while the third group is the copy number high, characterized by p53 mutations, and the fourth group entails the copy number low, with no specific surrogates [9,10]. Currently, the TGA molecular classification of EC has been replacing the classic categorization in type I and type II tumors.

Targeted sequencing to determine *POLE* mutations and the use of immunohistochemistry surrogates (*i.e.*, MMR and p53) have been commonly applied in clinical practice [11–13]. In light of the new molecular classification, G3 endometrioid endometrial carcinomas are the only ones represented in every molecular category of the TCGA classification. Several authors have shown that high-grade ECs with different molecular signatures behave heterogeneously, with patients who have *POLE*-ultramutated tumors showing a survival advantage [9,14]. The main objective of this systematic review was to determine the association between *POLE* mutational status, overall-survival (OS), and progression-free-survival (PFS) among patients with G3 endometrioid EC. We also aimed to determine the prevalence of *POLE* mutations in G3 endometrioid EC. To the best of our knowledge, this is the first systematic review and meta-analysis focusing on only the clinical outcomes of G3 endometrioid EC with *POLE* mutations.

## 2. Methods

### 2.1. Study protocol

This systematic review and meta-analysis was conducted according to the Preferred Reporting Items for Systematic Reviews and Meta-Analysis (PRISMA) statement [15] (see Supplementary data, Table S1 PRISMA 2020 checklist). The study was also preregistered with PROSPERO (No: CRD4202340008) [16].

### 2.2. Information sources and search strategy

A comprehensive review of the literature was performed on the 9th of March of 2023. The literature search was performed using the major electronic databases: PubMed/Medline, EMBASE, Cochrane Library, Scopus, and Web of Science. The search strategy (Supplementary data, Table S2) combined Boolean operators with the following search terms:

- Endometrial Cancer or Endometrial Carcinoma or EC
- High-Grade Endometrioid Endometrial Cancer or G3 Endometrioid Endometrial Cancer or G3 Endometrioid Endometrial Carcinoma
- *POLE* mutant or *POLE* mutation or Polymerase Epsilon mutation or *POLE* EDM mutation.

Only human studies were considered, and no restrictions were applied to the search in regard to language, year of publication, or study type. References of the most relevant studies and reviews were manually screened to identify any missing publications that were not retrieved by the electronic search. New searches were also performed to ensure inclusion of any eligible new publications during the performance of this review. Artificial intelligence software was used to store, organize, and manage all the references arising from the literature search [17].

### 2.3. Eligibility criteria and selection process

Only English manuscripts in which *POLE* mutation was tested by genetic sequencing with a clear statement of this information were considered eligible. Other inclusion criteria included:

- Adequate clinical and pathological data specifically regarding tumor grading and histology (only high-grade endometroid EC)
- Clear statement of oncologic outcomes (PFS and OS)
- Presentation of sufficient data allowing extraction of the hazard ratio (HR), and calculation of the standard error (ER), and the odds ratio (OR).

Published abstracts without published manuscripts, case reports (single), commentaries, letters to editors, editorials, and review articles (wrong publication type) were excluded. Articles were also discarded if they lacked enough data for calculation, lacked confirmation of *POLE* status determination by genetic sequencing, or had inconclusive data regarding either histology or tumor grading (wrong population). All duplicate studies were excluded. Two reviewers independently assessed all titles and abstracts of the retrieved search articles. The selection of full-text articles for inclusion was performed independently by two reviewers, and any disagreement was solved by a third independent reviewer.

### 2.4. Data collection process and data items

All studies were independently analyzed by two reviewers, and disagreements were resolved by a third independent reviewer. Data were extracted by two reviewers and evaluated by an additional reviewer. As applicable, the corresponding authors of the included studies were contacted to obtain or confirm data. Data on study population characteristics (including clinical and pathological data), OS, PFS, and prevalence (*POLE* mutation in G3 endometrioid EC) were extracted.

### 2.5. Risk of bias assessment

One reviewer independently assessed the quality of the studies and the risk of bias using the Quality in Prognosis Studies (QUIPS) tool, as recommended by the Cochrane Prognosis Methods Group [18]. A second reviewer reviewed this assessment, and disagreements were resolved by a third independent reviewer. The QUIPS tool includes the following six domains to evaluate the validity and bias in studies of prognostic factors: study participation, study attrition, prognostic factor measurement, outcome measurement, confounding factors, and study analysis and reporting [18]. Risk of bias was categorized as high, intermediate, or low [18]. Publication bias was assessed by inspecting funnel plots for each meta-analysis conducted.

### 2.6. Statistical analyses

For time-to-event data, we used the generic inverse variance method, pooled hazard ratios (HRs), and corresponding 95% confidence intervals (CIs). For each study, we used individual patient data (IPD) if available from the study team. If IPD were not available, we extracted information about time-to-event outcomes using methods described in the literature [19]. When we considered studies to be similar enough (in terms of participants, settings, intervention, and outcome measures) to allow pooling of data using meta-analysis, we assessed the degree of heterogeneity by visual inspection of forest plots. We estimated the percentage of heterogeneity between studies (which could not be ascribed to sampling variation, I^2^). When possible, subgroup analyses were also performed.

We estimated participant-level survival data from published Kaplan-Meier curves using validated algorithms by Guyot and colleagues [19]. Briefly, we downloaded, preprocessed, and digitized raster images of survivor curves to obtain their step function, including the step timings. If available, additional information such as number-at-risk tables and total number of events were used to further improve the calibration of the reconstruction algorithm. We then recovered time-to-event information on individual women by solving the inverted Kaplan-Meier product-limit equations. Comparisons of reconstructed curves and the original Kaplan-Meier curves demonstrated that the algorithms robustly recovered participant-level survival time from published studies.

We analyzed PFS and OS using both a one-stage method described by Guyot et al. (using reconstructed or original individual participant data) and a two-stage approach (prespecified inverse variance-weighted meta-analyses) [19]. For one-stage meta-analyses, we used the Kaplan-Meier method to calculate OS and PFS. We also used Cox-proportional hazards models to address between-study heterogeneity using a variety of approaches. We regarded the shared-frailty model to be the most robust approach as it most explicitly incorporates a gamma-distributed random-effects term to account for between-study heterogeneity. We calculated median follow-up times using the reverse Kaplan-Meier method.

Post-hoc sensitivity analyses were conducted for OS and PFS by including only data from trials using the reported aggregate-level data. For prevalence calculation, the total number of individuals screened was used as the denominator. Data were subjected to Freeman-Tukey transformation (double arcsine transformation) to avoid negative prevalence in the CI, which was limited to between 0 and 100%. For the analysis of publication bias, we conducted a linear regression of funnel plot asymmetry using Egger’s test. Statistical significance was considered at *p* < 0.05. R statistical software (version 4.3.0), package meta [20], was used for all statistical analyses.

## 3. Results

### 3.1. Study selection, characteristics of the included studies, and quality assessment

**Fig. 1.**
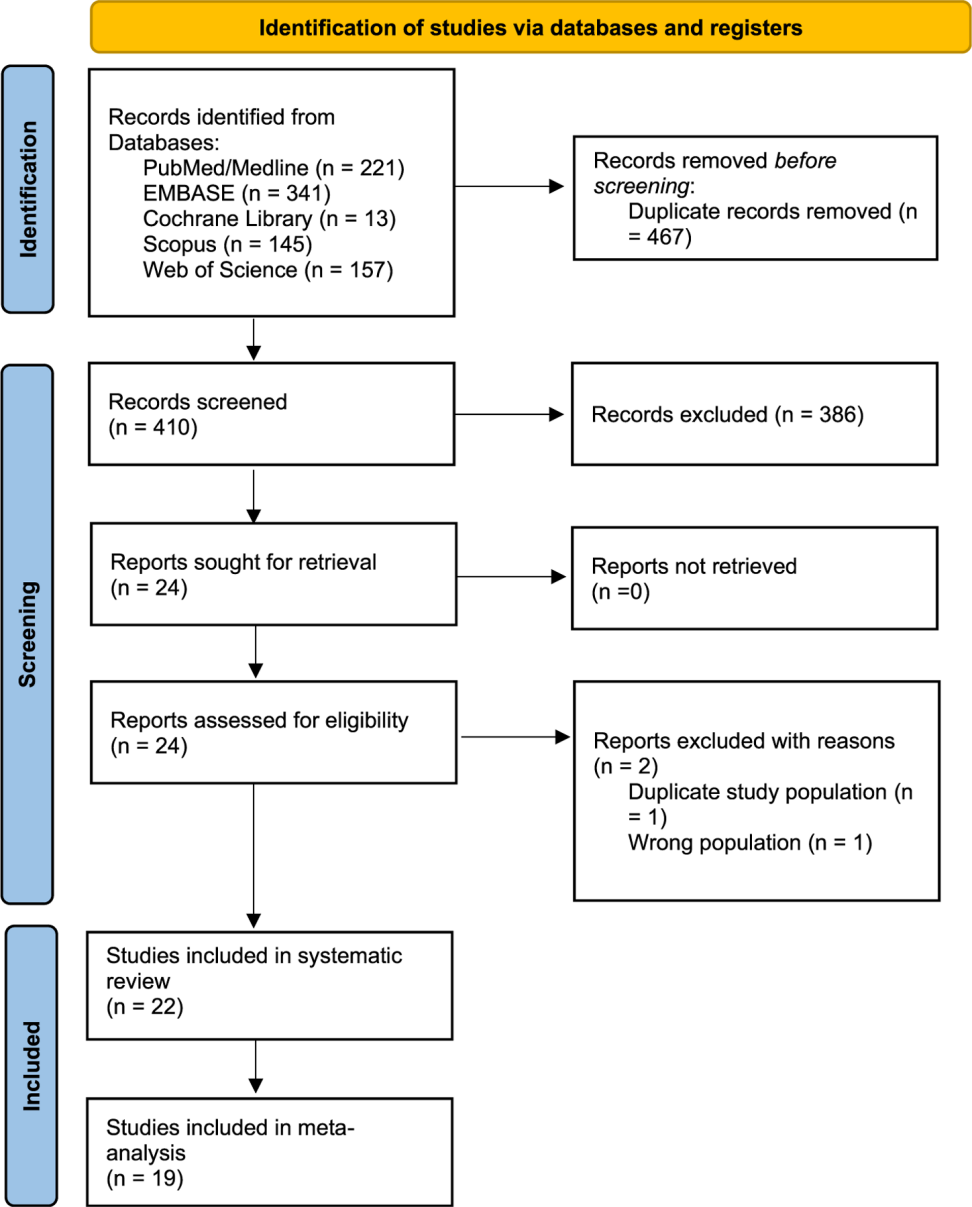
PRISMA 2020 flow diagram of systematic review process and study selection.

The search yielded 877 records, of which 410 were screened and 386 were excluded. The full texts of 24 articles were assessed for eligibility, and one of these studies was excluded due to a duplicate study population [21], while another was excluded due to inclusion of an inappropriate population [22] (Fig. 1). There were 22 studies [9,14,23–42] that met all inclusion criteria and were included in the systematic review (Fig. 1).

The characteristics of the included studies are presented in Table 1. The included articles were published between 2014 and 2023 and included a total of 3116 patients with high-grade endometrioid endometrial (Table 1). Table 2 shows the quality assessment results of the included studies according to the respective risk of bias. In the meta-analyses, only 19 studies with a total of 3092 patients were included after excluding 3 studies because they did not present enough data for quantitative syntheses [26,36,38].

### 3.2. Overall survival

**Fig. 2.**
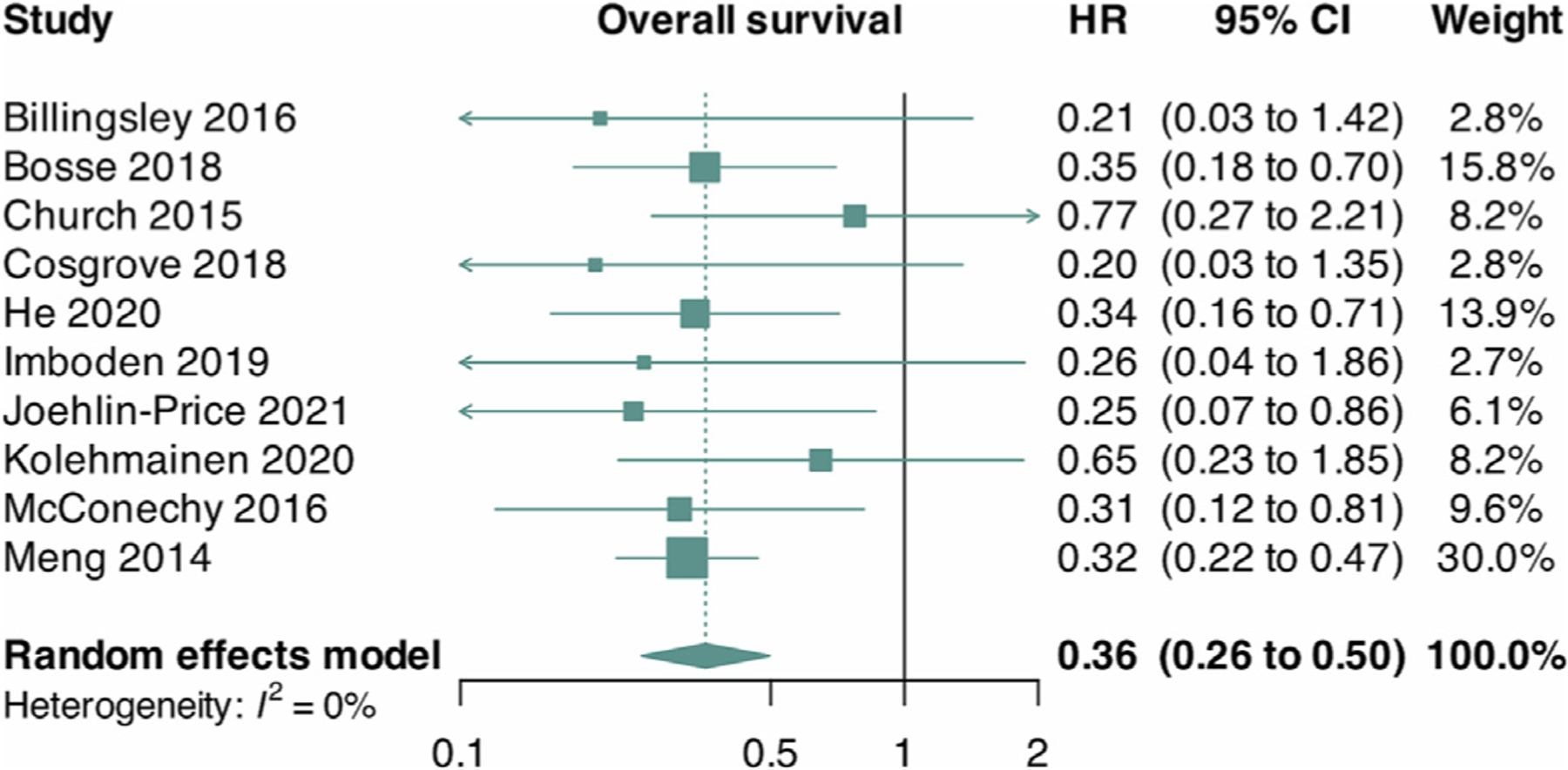
Forest plot for overall-survival. HR, hazard ratio; CI, confidence interval.

We pooled aggregate-level data from six trials and reconstructed approximate IPD from four trials. Overall, we found a lower risk of death among patients with *POLE* mutations when compared with patients without specific mutations (HR = 0.36, 95% CI 0.26 to 0.50, I^2^ = 0%, 10 trials) (Fig. 2). We conducted a post-hoc sensitivity analysis by including only data from trials using the reported aggregate-level data, which had overlapping results with the primary analysis (HR = 0.44, 95% CI 0.24 to 0.78, I^2^ = 50%, 6 trials) (Supplementary data, Fig. S1). We also conducted a linear regression of funnel plot asymmetry using Egger’s test, which did not indicate evidence of publication bias (*p* value = 0.97).

### 3.3. Progression-free survival

**Fig. 3.**
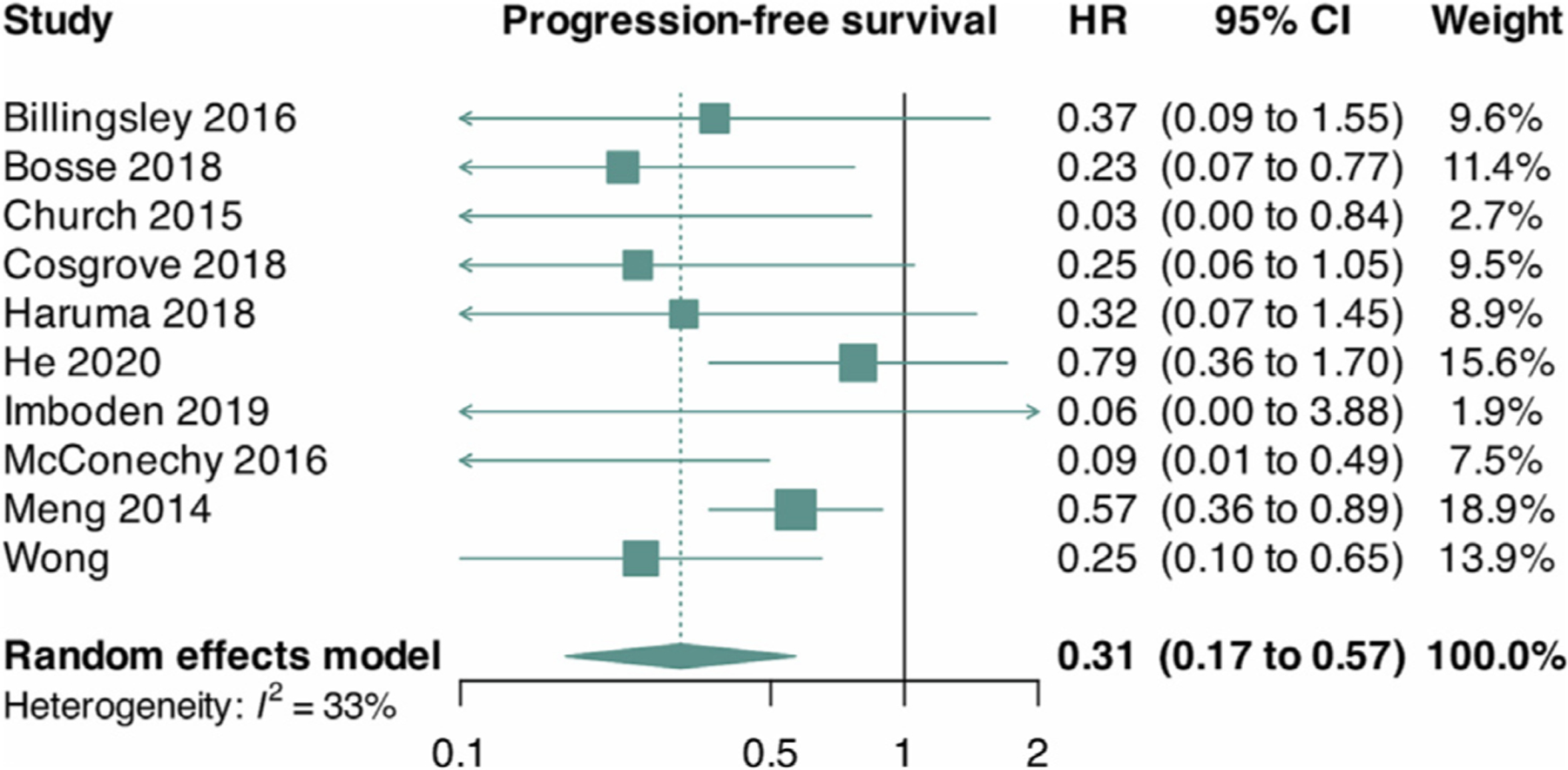
Forest plot for progression-free survival. HR, hazard ratio; CI, confidence interval.

We pooled aggregate-level data from seven trials and reconstructed approximate IPD data from three trials. Overall, we found a lower risk of disease progression among patients with *POLE* mutations when compared to patients of all other TCGA subgroups (HR = 0.31, 95% CI 0.17 to 0.57, I^2^ = 33%, 10 trials) (Fig. 3). We conducted a post-hoc sensitivity analysis by including only data from trials using the reported aggregate-level data, which showed overlapping results with the primary analysis (HR = 0.21, 95% CI 0.10 to 0.44, I^2^ = 0%, 7 trials) (Supplementary data, Fig. S2). We also conducted a linear regression of funnel plot asymmetry using Egger’s test, which suggested evidence of publication bias (*p* value = 0.004) (Supplementary data, Fig. S3).

### 3.4. Prevalence of POLE mutations

**Fig. 4.**
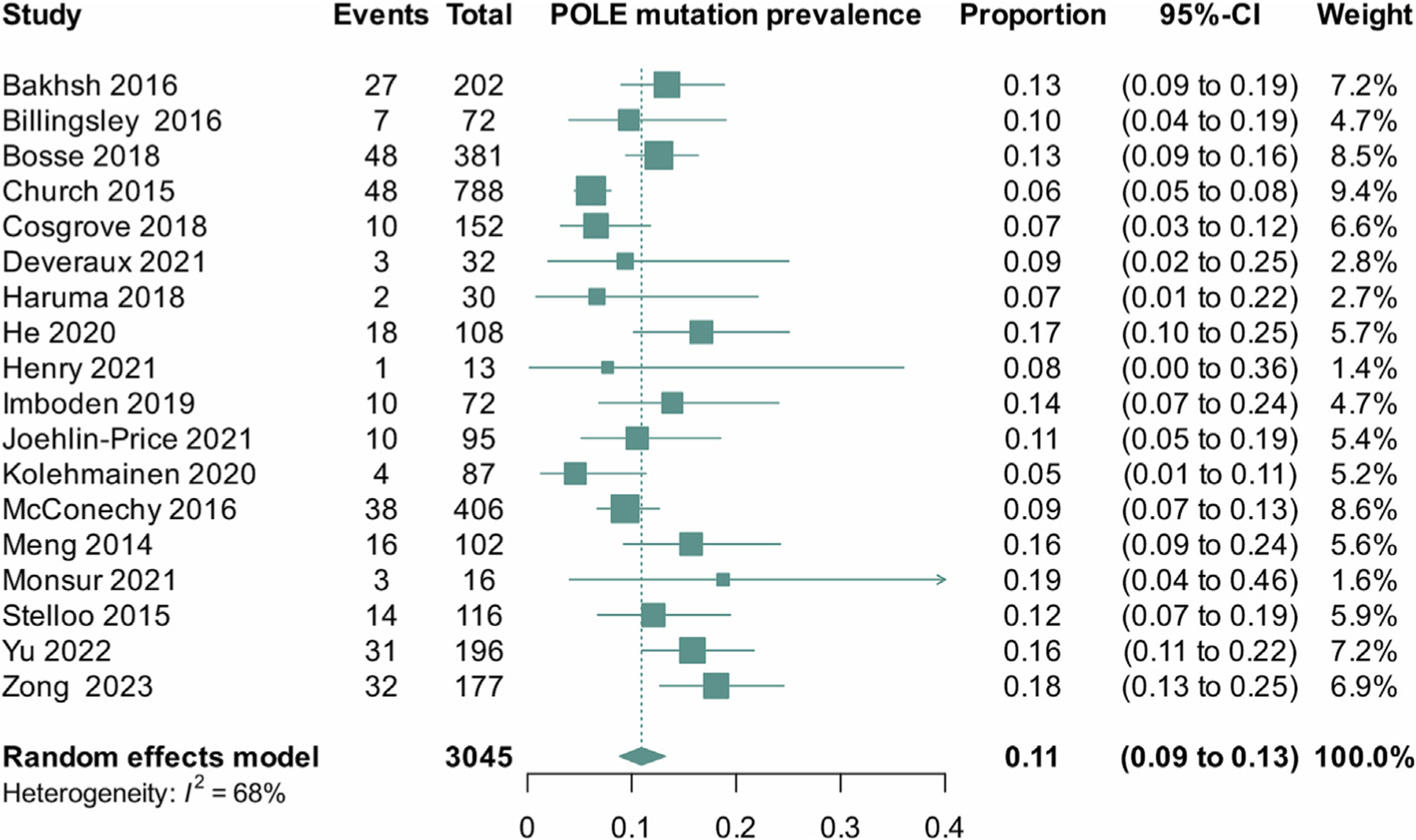
Prevalence of *POLE* mutation in G3 endometrioid endometrial cancer. CI, confidence interval.

The pooled calculated prevalence was 11% (95% CI 9% to 13%, I^2^ = 68%, 18 studies) (Fig. 4). We conducted a linear regression of funnel plot asymmetry using Egger’s test, which did not identify evidence of publication bias (p value = 0.11).

## 4. Discussion

To the best of our knowledge this is the first systematic review and meta-analysis addressing oncologic outcomes of G3 (high-grade) endometrioid EC with *POLE* mutations. *POLE* is a gene involved in DNA replication and repair. As described previously, *POLE* mutations are associated with high tumor mutation burden, which may trigger the immune system to recognize the cancer cells as foreign and mount a robust anti-tumor response [10,37]. This could partially explain why *POLE*-ultramutated tumors have a more favorable prognosis. As mentioned, G3 endometrioid EC constitutes a heterogenous subtype of EC [9], so patients with these tumors need a more tailored approach to avoid subjecting them to unnecessary adjuvant therapy. As studies such as the PORTEC 4a study are still underway, there is a need to start integrating the molecular profiling of these tumors in clinical settings [13,43].

Our review indicated not only a survival advantage in G3 *POLE*-mutated endometrioid EC, but also an increased PFS. These findings provide extra strength to the literature indicating that *POLE*-ultramutated tumors are in fact a specific subtype of endometrial carcinomas, irrespective of the tumor grading. With the new FIGO classification of EC already published, we hope that this review may contribute to emphasizing the need for a tailored approach in terms of adjuvant therapy for the specific combination of high grade endometrioid EC with *POLE mutations*.

This study had several strengths. Firstly, we included a high number of studies and patients and used studies from different populations, which increases the generalizability of the results. Secondly, our results regarding the pooled estimates of both OS and PFS with aggregate-level data and reconstructed approximate IPD were highly consistent. The sensitivity analyses showed stable results with the same direction and magnitude of the pooled estimates when compared to the primary analyses. Thirdly, this meta-analysis is strengthened by the overall good quality of the individual studies included. Importantly, by summarizing the available data regarding the more favorable prognostic outcomes of high-grade endometrioid EC with *POLE* mutations, we provide clinicians with another perspective to discuss management with patients while data from multicentric studies are still pending.

However, there are still limitations in this work. Firstly, only retrospective studies and no randomized control trials were included in our review.

We acknowledge that the observed survival advantage may also be related to adjuvant therapy that was given to patients in the evaluated studies. However, one can argue that due to the more favorable natural history of this subtype of tumors, all early-FIGO-stage tumors could probably be safely managed without adjuvant therapy. Further research, highlighting which patients can be safely managed without adjuvant therapy is warranted. Additionally, based on the funnel plot for PFS, there is an underrepresentation of studies with a higher HR of disease progression, which may reflect publication bias. We also acknowledge that the conclusions of our study are general, and they cannot be applied to the individual patient, as further research is needed to determine if de-escalation of adjuvant therapy is safe in POLE high-grade EC.

From a future perspective, we hope that this review will encourage researchers to conduct further studies to address the oncologic safety of omitting adjuvant therapy among patients with G3 endometrioid EC. In conclusion, our data support that *POLE* mutations in high-grade endometrioid ECs are associated with a more favorable prognosis with increased OS and PFS.

## Supplementary Material

MMC4

MMC5

MMC3

MMC1

MMC2

## Figures and Tables

**Table 1:** Characteristics of the included studies.

Study Author	Year of Publication	Country	Study type	G3 Cohort Size	POLE Mutant number	Sequencing Methodology	Outcomes (OR and PFS)
Bakhsh et al.	2016	USA, Canada	Retrospective observational study	202	27	–	OS: no data PFS: no data
Billingsley et al.	2016	USA	Retrospective observational study	72	7	PCR amplification and Sanger sequencing	OS: Adjusted HR 0.19 (95% CI, 0.03–1.42) PFS: Adjusted HR 0.37 (98% CI, 0.09–1.55)
Bosse et al.	2018	USA/Netherlands/Canada/Spain/UK	Retrospective observational study	381	48	Sanger Sequencing or NGS	OS: HR 0.36 (95% CI, 0.18–0.70) (Uni); HR 0.56 (95% CI, 0.27–1.15) (Multi) PFS: HR 0.17 (95% CI, 0.05–0.54) (Uni); HR 0.23 (95% CI, 0.07.0.77) (Multi)
Church et al.	2015	UK, Netherlands	Retrospective observational study	788	48	Sanger Sequencing	OS: Adjusted HR 1.06 (95% CI, 0.59–1.92) PFS: Adjusted HR 0.11 (95% CI, 0.001–0.84)
Cosgrove et al.	2018	USA	Retrospective observational study	152	10	NGS	OS: G3 HR 3.82 (95% CI, 2.48–5.89; p < 0.001); POLE HR 0.22 (95% CI, 0.03–1.57; *p* = 0.129) (Univariate analysis); G3 HR 2.76 (95% CI, 1.65–4.60; *p* < 0.001) (multi); POLE HR 0.19 (95% CI, 0.03–1.35; *p* = 0.096) PFS: G3 HR 3.02 (95% CI, 2.09–4.34; p < 0.001); POLE HR 0.27 (95% CI, 0.07–1.10; *p* = 0.068) (Univariate analysis); G3 HR 2.25 (95% CI, 1.46–3.47; p < 0.001) (Multi); POLE HR 0.26 (95% CI, 0.06–1.05; *p* = 0.059) (Multi)
Dai et al.	2022	China	Retrospective observational study	2	1	NGS	OS: no data PFS: no data
Devereaux et al.	2021	USA	Prospective study	32	3	SNaPshot (PCR amplification and multiplexed single-nucleotid primer)	OS: no data PFS: no data
Haruma et al.	2018	Japan	Retrospective observational study	30	2	Sanger Sequencing	OS: no data PFS: For ECs with POLE-mutations, MSI and non-MSI, five-year PFSs were 100%, 89.5%, and 74.5% (*p* = 0.0420), five-year ECSs were 100%,88.7%, and 84.5% (*p* = 0.3162), respectively
He et al.	2020	China	Retrospective observational study	108	18	PCR amplification and Sanger sequencing	OS: no data PFS: G3 HR 1.28 (95% CI, 1.14–1.43; p < 0.001); POLE Mut HR 3.25 (95% CI, 0.34–31.3; p = 0.31); POLE wild-type HR 1.27 (95% CI, 1.14–1.42; p < 0.001)
Henry et al.	2021	New Zealand	Retrospective observational study	13	1	NGS	OS: no data PFS: no data
Imboden et al.	2019	Switzerland/Sweden	Retrospective observational study	72	10	Sanger Sequencing	OS: HR 0.258 (CI, 0.036–1.862; *p* = 0.179) All POLE Mut PFS: Cox-regression analysis for risk of recurrence, no significance was reached (CI, 0.001–3.884; *p* = 0.172). In addition, analysis of the non-endometrioid tumors (*N* = 98) showed that the POLE mutation (*N* = 7) did not have a significant positive effect on survival.
Joehlin-Price et al.	2021	USA	Retrospective observational study	95	10	PCR amplification and NGS	OS: *p* = 0.082 (95% CI) PFS: *p* = 0.526 (95% CI)
Kolehmainen *et al*	2020	Finland	Retrospective observational study	87	4	NGS	OS: no data PFS: no data
McConechy et al.	2016	Canada	Retrospective observational study	406	38	Sanger Sequencing	OS: no data PFS: HR(F) 0.135 (95% CI, 0.015–0.495)
Meng et al.	2014	Canada	Retrospective observational study	102	16	PCR amplificationswere performed as previously described using 50 ng genomic DNA and the primer sets using High-Fidelity Tag DNA polymerase	OS: no data PFS: no data
Miller et al.	2020	USA	Retrospective observational study	12	6	NGS	OS: no data PFS: no data
Monsur et al.	2021	Japan	Retrospective observational study	16	3	PCR amplification and Sanger sequencing	OS: no data PFS: no data
Stasenko et al.	2020	USA	Retrospective observational study	10	10	NGS	OS: no data PFS: no data
Stelloo et al.	2015	UK, Netherlands, France	Retrospective observational study	116	14	Sanger Sequencing	OS: no data PFS: no data
Wong et al.	2016	Singapore	Retrospective observational study	47		Sanger Sequencing & NGS	OS: no deaths PFS: no recurrence
Yu et al.	2022	China	Retrospective observational study	196		WES and Sanger sequencing	OS: 96.6% PFS: 97.7%
Zong et al.	2023	China	Retrospective observational study	177		PCR amplification and Sanger sequencing	OS: Kapplan B PFS:Kapplan A

CI, confidence interval; HR, hazard ratio; NGS, next-generation sequencing; OS, overall-survival; PCR, Polymerase chain reaction; PFS, progression-free survival; WES, whole exome sequencing.

**Table 2:** Methodological quality assessment according to Quality in Prognostic Studies (QUIPS) tool.

Study	Study participation	Study attrition	Prognostic factor measurement	Outcome measurement	Study confounding	Study analysis and reporting
Bakhsh *et al.* (2016)	●	●	●	●	●	●
Billingsley *et al.* (2016)	●	●	●	●	●	●
Bosse *et al.* (2018)	●	●	●	●	●	●
Church *et al.* (2015)	●	●	●	●	●	●
Cosgrove *et al.* (2018)	●	●	●	●	●	●
Dai *et al.* (2022)	●	●	●	●	●	●
Devereaux *et al.* (2021)	●	●	●	●	●	●
Haruma *et al.* (2018)	●	●	●	●	●	●
He *et al.* (2020)	●	●	●	●	●	●
Henry *et al.* (2021)	●	●	●	●	●	●
Imboden *et al.* (2019)	●	●	●	●	●	●
Joehlin-Price *et al.* (2021)	●	●	●	●	●	●
Kolehmainen *et al.* (2021)	●	●	●	●	●	●
McConechy *et al.* (2016)	●	●	●	●	●	●
Meng *et al.* (2014)	●	●	●	●	●	●
Miller *et al.* (2020)	●	●	●	●	●	●
Monsur *et al.* (2021)	●	●	●	●	●	●
Stasenko *et al.* (2020)	●	●	●	●	●	●
Stelloo *et al.* (2015)	●	●	●	●	●	●
Wong *et al.* (2016)	●	●	●	●	●	●
Yu *et al.* (2022)	●	●	●	●	●	●
Zong *et al.* (2023)	●	●	●	●	●	●

Green: low risk of bias; yellow: moderate risk of bias.

## Data Availability

All data relevant to the study are included in the article. Further information can be obtained from the corresponding author.

## HIGHLIGHTS

- Systematic review and meta-analysis of 19 studies (total of 3092 patients) with high-grade endometrioid endometrial cancer.
- We found a lower risk of disease progression and death in patients with *POLE* mutations.
- We estimated a pooled prevalence of POLE mutation of 11% (95% CI 9% to 13%, I^2^ = 68%).
