# Supplementary material for: Prognosis of polymerase epsilon (POLE) mutation in high-grade endometrioid endometrial cancer: Systematic review and meta-analysis
Source: Gynecol Oncol. Author manuscript; Available in PMC 2024 Jul 31. (PMC11290341; doi:10.1016/j.ygyno.2024.01.018)
Supplement: MMC5 [file NIHMS2007031-supplement-MMC5.docx]

**Table S2.** Retrieval search strategy.

| **Query** | **Search** |
| --- | --- |
| **PubMed/Medline** |  |
| #1 | "endometrial cancer*" OR "endometrial carcinoma*" OR EC OR "high-grade endometrioid endometrial cancer*" OR "G3 endometrioid endometrial cancer*" OR "G3 endometrioid endometrial carcinoma*" |
| #2 | "POLE mutant*” OR "POLE mutation*" OR "Polymerase Epsilon mutation*" OR "POLE EDM mutation*" |
| #3 | #1 AND #2 |
| **EMBASE** |  |
| #1 | "endometrial cancer*" OR "endometrial carcinoma*" OR EC OR "high-grade endometrioid endometrial cancer*" OR "G3 endometrioid endometrial cancer*" OR "G3 endometrioid endometrial carcinoma*" |
| #2 | "POLE mutant*” OR "POLE mutation*" OR "Polymerase Epsilon mutation*" OR "POLE EDM mutation*" |
| #3 | #1 AND #2 |
| **Cochrane Library** |  |
| #1 | "endometrial cancer*" OR "endometrial carcinoma*" OR EC OR "high-grade endometrioid endometrial cancer*" OR "G3 endometrioid endometrial cancer*" OR "G3 endometrioid endometrial carcinoma*" |
| #2 | "POLE mutant*” OR "POLE mutation*" OR "Polymerase Epsilon mutation*" OR "POLE EDM mutation*" |
| #3 | #1 AND #2 |
| **Scopus** |  |
| #1 | "endometrial cancer*" OR "endometrial carcinoma*" OR EC OR "high-grade endometrioid endometrial cancer*" OR "G3 endometrioid endometrial cancer*" OR "G3 endometrioid endometrial carcinoma*" |
| #2 | "POLE mutant*” OR "POLE mutation*" OR "Polymerase Epsilon mutation*" OR "POLE EDM mutation*" |
| #3 | #1 AND #2 |
| **Web of Science** |  |
| #1 | "endometrial cancer*" OR "endometrial carcinoma*" OR EC OR "high-grade endometrioid endometrial cancer*" OR "G3 endometrioid endometrial cancer*" OR "G3 endometrioid endometrial carcinoma*" |
| #2 | "POLE mutant*” OR "POLE mutation*" OR "Polymerase Epsilon mutation*" OR "POLE EDM mutation*" |
| #3 | #1 AND #2 |

EDM, exonuclease domain mutations; G3, grade 3; POLE, Polymerase Epsilon.
