# Supplementary figures and images for: Prognosis of polymerase epsilon (POLE) mutation in high-grade endometrioid endometrial cancer: Systematic review and meta-analysis
Source: Gynecol Oncol. Author manuscript; Available in PMC 2024 Jul 31. (PMC11290341; doi:10.1016/j.ygyno.2024.01.018)

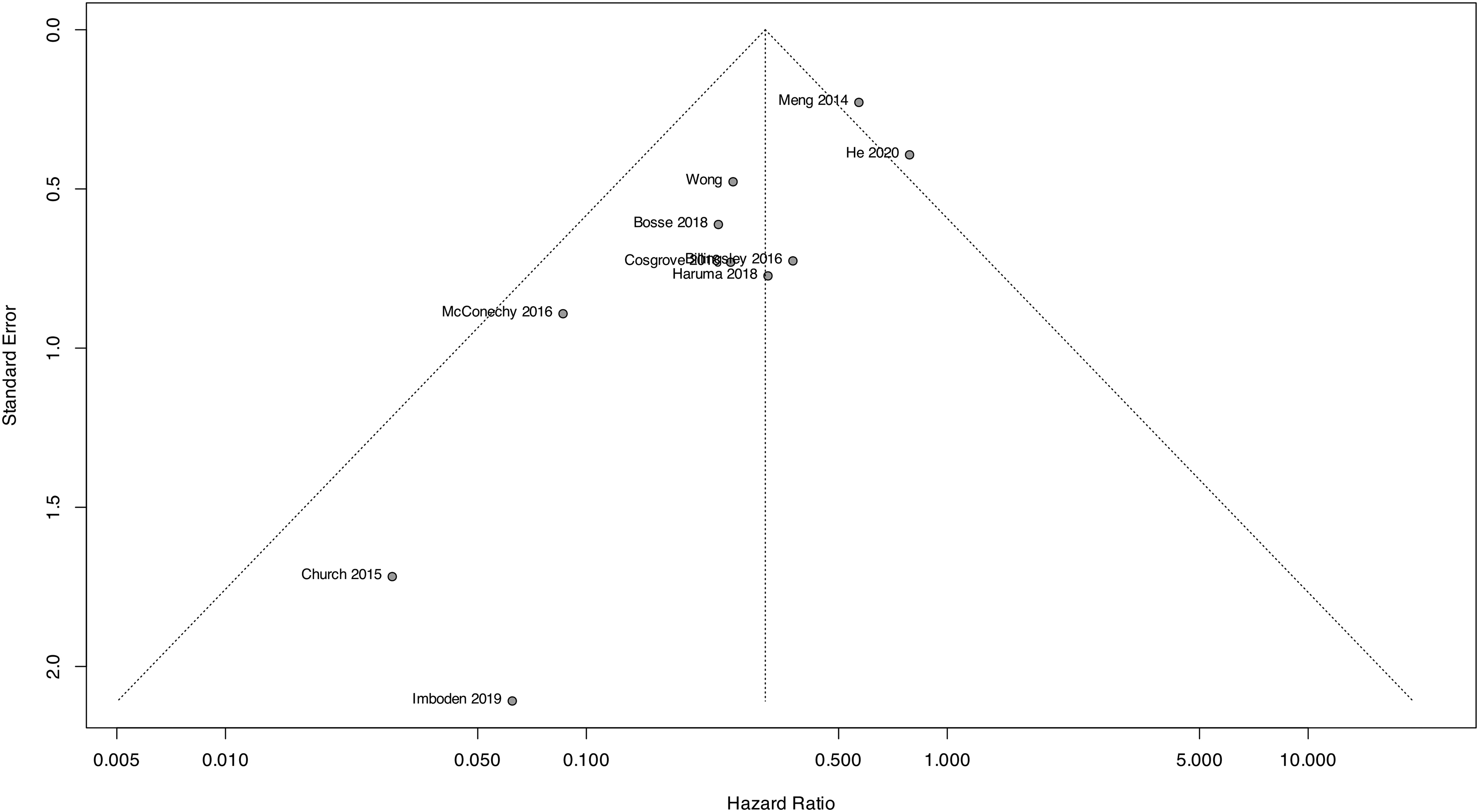

Supplement: MMC3 [file NIHMS2007031-supplement-MMC3.jpg]

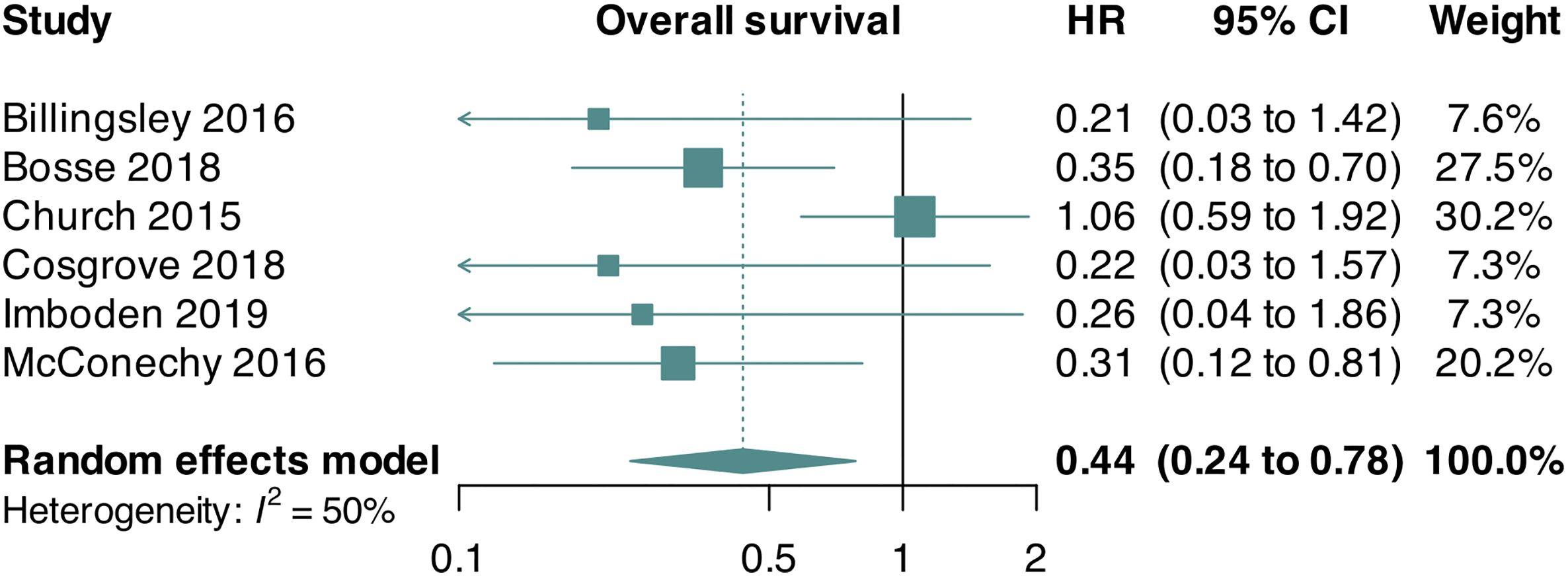

Supplement: MMC1 [file NIHMS2007031-supplement-MMC1.jpg]

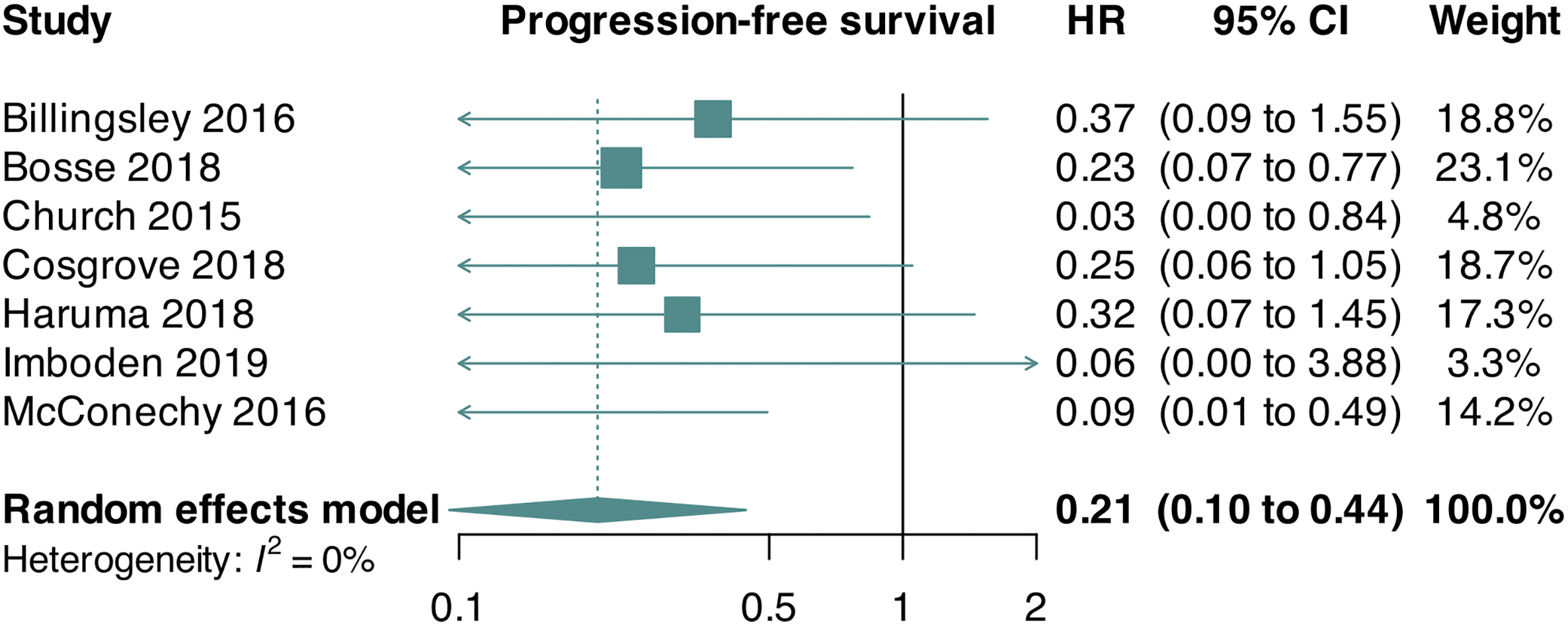

Supplement: MMC2 [file NIHMS2007031-supplement-MMC2.jpg]
